# Collaborative representation-based classification of microarray gene expression data

**DOI:** 10.1371/journal.pone.0189533

**Published:** 2017-12-13

**Authors:** Lizhen Shen, Hua Jiang, Mingfang He, Guoqing Liu

**Affiliations:** 1 School of Biotechnology and Pharmaceutical Engineering, Nanjing Tech University, Nanjing, 211800, China; 2 School of Physical and Mathematical Sciences, Nanjing Tech University, Nanjing, 211800, China; Harbin Institute of Technology Shenzhen Graduate School, CHINA

## Abstract

Microarray technology is important to simultaneously express multiple genes over a number of time points. Multiple classifier models, such as sparse representation (SR)-based method, have been developed to classify microarray gene expression data. These methods allocate the gene data points to different clusters. In this paper, we propose a novel collaborative representation (CR)-based classification with regularized least square to classify gene data. First, the CR codes a testing sample as a sparse linear combination of all training samples and then classifies the testing sample by evaluating which class leads to the minimum representation error. This CR-based classification approach is remarkably less complex than traditional classification methods but leads to very competitive classification results. In addition, compressive sensing approach is adopted to project the high-dimensional gene expression dataset to a lower-dimensional space which nearly contains the whole information. This compression without loss is beneficial to reduce the computational load. Experiments to detect subtypes of diseases, such as leukemia and autism spectrum disorders, are performed by analyzing the gene expression. The results show that the proposed CR-based algorithm exhibits significantly higher stability and accuracy than the traditional classifiers, such as support vector machine algorithm.

## Introduction

The development of microarray technology facilitates the collection of information containing expression values of multiple genes under different experimental conditions. This technology also promotes and affects the progress in biological and biomedical research [1–3]. Therefore, huge amounts of genome-wide expression data have been remarkably acquired, and the quantity of data is continuously growing. These data provides the opportunity to better understand the tissues being studied and explore a finer molecular distinction between health states. Thus, approaches based on machine learning, which can automatically acquire qualitatively interesting patterns from gene data, have been widely adopted [4–7]. Among these machine learning pathways, support vector machine (SVM) and K-nearest neighbor (KNN) are used to study performance [8–11]. To facilitate a more flexible and comprehensive analysis, Pse-in-one and Pse-analysis have been proposed. These methods are considered powerful bioinformatics analysis tools based on web server and Python package, respectively [12, 13]. These tools can generate any desired pseudo components or feature vectors for protein/peptide and DNA/RNA sequences according to the need of users’ in their studies. In [14], various neural network technologies for cancer classification were surveyed, and the results are beneficial to exploit the most cost-effective approaches for clinicians. Zheng et al. [15] used independent component analysis to refine a subset of genes to further improve the clustering performance of nonnegative matrix factorization. However, gene data are always in high-dimensional space because of the enormous numbers of measurements (e.g., genes and probes). The high-dimensional characteristic limits the applicability of the majority of conventional classifier models. This characteristic also leads to the poor performance of conventional models in the identifying diseases from genome-wide expression data. Moreover, parameters should be optimized to facilitate the implementation of the algorithm depending on the structure of the data set.

Sparse representation is well-known as a powerful tool in various applications and has been highly developed for signal processing and machine learning [16]. Sparse representation-based classification (SRC) was introduced to identify gene expression without requiring any parameter optimization [17]. The SRC scheme has been successfully applied in the diagnosis of microarray gene expression in cancer. In [18], SRC was applied and showed better performance than the state-of-the art methods in protein fold recognition. In the SRC scheme, a microarray gene expression y can be expressed as y = Φ*α* by sparse representation over a dictionary Φ, while *α* is a sparse vector. Assuming that *l*_0_-norm indicates the number of non-zeros in *α*, the sparsity of *α* can be represented by *l*_0_-norm. Thus, *α* can be evaluated with the criteria of *l*_0_-norm minimization. However, the solution of *l*_0_-norm minimization is NP-hard, which is very time-consuming. Consequently, *l*_1_-norm minimization is considered as the alternative to *l*_0_-norm minimization because of its closest convex function of the former. The former minimization process is widely equipped with in sparse coding as follows: min_*α*_‖*α*‖_1_ s.t. ‖y − Φ*α*‖_2_ ≤ *ε*, where *ε* is a small constant. Although the computational complexity of *l*_1_-norm minimization is much lower than that of *l*_0_-norm minimization, the applications of *l*_1_-norm minimization is still limited by the high complexity in real-time scenarios. Therefore, various algorithms have been presented to accelerate *l*_1_-norm minimization.

Another issue is that the dimension of the feature space may be too high for classification algorithm. Numerous algorithms may become invalid or infeasible when lots of feature data in a dimension have to be processed [19–21]. A successful method is to extract a small number of discriminative information from a high-dimensional space. That is, high-dimensional data can be mapped to a feature space with lower dimension to facilitate the design of a classification algorithm. The algorithm of feature extraction is effective but highly complex. Compressive sensing (CS) theory is a well-known solution for sparse signals in sampling theory [22]. The main result of CS, which was introduced by Candés and Tao [23] and Donoho [24], is that a length-*N* signal *x*, that is, *K*-sparse in some basis can be recovered exactly in the polynomial from just *M* = *O*(*K* log(*N*/*K*)) linear measurements of the signal. Using the CS approach, high-dimensional feature data can be simply projected to a space with much lower dimension with a random sensing matrix. The low-dimensional data retain enough information and can recover the original high-dimensional features. In this paper, we employ a measurement matrix which is assumed to be very sparse to efficiently compress the feature from the gene expression dataset. CS facilitates the extraction of the feature data to a low-dimensional one in the compressed domain.

To improve the sparse representation with computationally expensive *l*_1_-norm minimization, this paper proposes a collaborative representation (CR)-based classification (CRC) to subtype gene expression datasets. CR collaboratively uses whole training samples from all classes to constitute the query sample y. CRC with regularized least square can achieve competitive classification results with considerably less complexity. Our works are beneficial in augmenting the study of sparsity-based genome-wide data pattern classification. We used benchmark cancer, autism spectrum disorder (ASD), and brain data sets, in the experiments. To measure the effectiveness of the CRC algorithm, accuracy in terms of prediction or error rate in classifying a selected gene subset is evaluated.

## Materials and methods

In this study, the classification problem can be depicted as follows: The microarray gene data sets, which serve as sample to train, are classified for the diagnosis and prognosis of various types of diseases. To apply sparse representation model, the coding vector of y should be sparse. Moreover, coding should be performed collaboratively over the whole dataset instead of each subset. By identifying the sparsest representation of y over *X*, the subset is naturally discriminative.

### Collaborative representation-based classification

Classification of gene expression datasets using algorithms has been well studied for the robust diagnosis and prognosis of various diseases to achieve high prediction accuracy [25]. The primary goal of this approach is to identify the important pathways or genes strongly associated with the clinical outcomes of interest. Sparse representation has been applied to classify genomic data [26, 27]. The corresponding performance was tested by distinguishing two subtypes of leukemia by analyzing microarray gene expression. With the selected leukemia genes from the 7129 ones, SRC achieved a classification accuracy of 97.4% when performed with the leave-one-out (LOO) method. When the tests were imposed on the same datasets, compared with existing methods, such as weighted vote, SVM, sparse logistic regression method, and rough sets method, SRC shows potential advantages with respect to improved classification rates with fewer informative genes. SRC also helps reduce computational load in terms of computational complexity and memory storage when processing high-dimensional data. Detecting subtypes of diseases, such as leukemia, according to different genetic markups is important. SRC is favorable for personalized therapy and improving treatment.

According to the theory of sparse representation, a complete dictionary of atoms, denoted by $\Phi \in R^{m\times n}$, can accurately represents any signal $x\in R^{m}$ by linearly combining these atoms in Φ. Nevertheless, if Φ is orthogonal, the representation of *x* is required with many atoms from Φ, leading to a complex computation. Then, the orthogonality of Φ is relaxed to allow less atoms to represent *x*. That is, more atoms is required to be involved in Φ to allow more choices to represent *x*. The dictionary Φ with redundant atoms is referred to as an over-complete matrix, leading to a sparser representation of signal *x*. Such sparse representation has been utilized in image restoration, in which great success has been achieved.

A total number of *K* classes of targets are assumed. Let $X_{i}\in R^{m\times n}$ denote the dataset of the *i*^*th*^ class, *X* = [*X*_1_, *X*_2_, ⋯, *X*_*K*_] and the column of *X*_*i*_ is a sample belonging to class *i*. The existence of a microarray gene data $y\in R^{m}$ is assumed, which can be coded as y = *Xα* + *w*, where *w* is a vector indicating the residual, *α* = [*α*_1_; ⋯; *α*_*i*_; ⋯; *α*_*K*_] and *α*_*i*_ denotes the coding vector corresponding to class *i*. If y belongs to the *i*th class, *w* is a zero vector, then y = *X*_*i*_*α*_*i*_ holds. Only the coefficients in *α*_*i*_ are significant when most entries in *α*_*j*_, *j* ≠ *i* are nearly zeros. That is, vector *α* is sparse, and its non-zero entries are used to encode the identity of sample y. Table 1 shows the matrix measurements of the microarray gene expression data for samples of prostate tumors and adjacent prostate tissue without tumor. Assuming that the training samples consist of these samples, the dictionary of samples is as follows:

$$\begin{array}{c} X=[\begin{array}{cccccc} 9 & -1 & -1 & -2 & -9 & 0\\ -11.4 & 17 & -1 & 0 & -19 & 0\\ 2.7 & 0 & 0 & -1 & 0 & 0\\ 0.6 & 3 & -1 & -2 & 0 & -2\\ 4.3 & 6 & 3 & 6 & 76 & 2\\ 28 & -6 & 0 & 3 & 9 & 0\\ \vdots & \vdots & \vdots & \vdots & \vdots & \vdots \\ 37.3 & 14 & 26 & 25 & -21 & 21 \end{array}]. \end{array}$$ (1)

A sample to be identified is treated as the measurement output, and *α* is to be reconstructed.

**Table 1:** Gene expression data.

	G1	G2	G3	G4	G5	G6	⋯	Gn	Label
X1	9	-11.4	2.7	0.6	4.3	28	⋯	37.3	Normal
X2	-1	17	0	3	6	-6	⋯	14	Cancer
X3	-1	-1	0	-1	3	0	⋯	26	Normal
X4	-2	0	-1	-2	6	3	⋯	25	Cancer
X5	-9	-19	0	0	76	9	⋯	-21	Normal
X6	0	0	0	-2	2	0	⋯	21	Cancer

Microarray gene expression datasets, such as breast cancer dataset, usually suffer from high dimensionality. In traditional SRC, enough training samples for each class is required for dictionary *X*_*i*_ to be over-complete. However, the sample size of the gene expression data is very small compared to its high dimensionality, leading to the under-complete matrix *X*_*i*_. Consequently, the representation error would be unacceptable, even when y belongs to class *i*. This characteristic leads to a failure decision made by the classifier. One obvious solution is to use more samples of class *i* to represent y. However, collecting huge number of samples is expensive. Nevertheless, differences among genes are very small such that the diverse classes of gene expression data share similarities. Thus, the testing gene *y* can be coded collaboratively by the dictionary of all samples *X* = [*X*_1_, *X*_2_, ⋯, *X*_*k*_] under the *l*_1_-norm sparsity constraint.

When y is collaboratively represented with all classes containing all samples, y can be classified individually by SRC by checking class by class [28]. The sparse solution to y = X*α* can be determined by solving the following optimization problem:

$$\begin{array}{c} \hat{\alpha }=\arg\min{∥\alpha ∥}_{0}\;s.t.\;y=X\alpha \end{array}$$ (2)

However, solving *l*_0_-optimization is an NP hard problem. Optimizations of *l*_0_-optimization and *l*_1_-optimization have recently been proved to be equivalent when *α* is sparse enough. In general, the sparse representation problem can be expressed as follows:

$$\begin{array}{c} \hat{\alpha }=\arg\min_{\alpha }{∥\alpha ∥}_{1}\;s.t.\;y=X\alpha \end{array}$$ (3)

We use sparsity constraint of *l*_2_-norm instead of that of *l*_1_-norm, Thus. the problem becomes as follows:

$$\begin{array}{c} \hat{\alpha }=\arg\min_{\alpha }{∥y-X\alpha ∥}_{2}^{2}+\lambda {∥\alpha ∥}_{1} \end{array}$$ (4)

If *X* is over-complete, $\hat{\alpha }$ is computed by an algorithm such as OMP algorithm. Then, y is perpendicularly projected onto the space spanned by *X*, which can be expressed as follows:

$$\begin{array}{c} \hat{y}=\sum_{i}X_{i}{\hat{\alpha }}_{i} \end{array}$$ (5)

In SRC, a new sample y_*new*_ can be classfied by evaluating the reconstruction representation errors:

$$\begin{array}{c} e_{i}=\arg\min_{i}{∥y_{new}-X_{i}{\hat{\alpha }}_{i}∥}_{2}^{2} \end{array}$$ (6)

where ${\hat{\alpha }}_{i}$ designates the coding coefficient vector drawn according to class *i*. When the number of classes is too large, a stable solution of the least square $\hat{\alpha }=\arg\min_{\alpha }{∥y-X\alpha ∥}_{2}^{2}$ cannot be obtained. To overcome this limitation, CRC is proposed to collaboratively represent the query sample using *X*. The corresponding *l*_2_-minimization with regularized least square is as follows:

$$\begin{array}{c} \hat{p}={\left(X^{T}X+\lambda I\right)}^{-1}X^{T}\;y \end{array}$$ (7)

Assuming *P* = (*X*^*T*^*X* + λ*I*)^−1^ *X*^*T*^, this formula shows that *P* is independent of y. Hence, *P* is only required to be computed once and can be shared in classifying different samples. The reduction in the complexity of CRC is not achieved at the expense of performance. Similar to SRC, $\hat{p}$ is used to classify the type of gene expression data. The representation residual $∥y-X_{i}{\hat{p}}_{i}{∥}_{2}$ is the main criteria for classification, where ${\hat{p}}_{i}$ is the coding vector associated with class *i*. However, the *l*_2_-norm $∥{\hat{p}}_{i}{∥}_{2}$ is also beneficial in providing some information about the class features of gene expression data and can be combined for classification. Such combination is useful to slightly improve the classification accuracy compared with that using only the representation residual.

### Compressive sensing-based dimensionality reduction

Gene expression datasets may have very high dimensionality. High-dimensional gene expression datasets always result in high computational load and degradation of model performance of classification algorithm [10, 19, 21]. Therefore, feature selection approach or dimensionality reduction methods should eliminate redundant and irrelevant feature data to decrease the ratio of features to samples. On the other hand, this process reduces the probability of overfitting.

A common method of dimensionality reduction in high-dimensional classification is choosing some data from a dataset in an order and dropping the other data. This approach would inevitably result in the loss of some information. Many probes in traditional microarrays are inactive during detection. That is, the microarray gene expression data can be sparse. Only a few significant entries of gene expression data matrix may be of interest. This phenomenon suggests the fast transformation of a microarray along with the CS measurement process, where each measurement *y* is a linear combination of entries in the microarray gene expression data vector *x*. A number of *N* gene expression data is assumed in every sample, but that at most *K* samples are collected, with *K* ≪ *N*. In random projection, a sensing matrix *R* with rows having unit length projects data from the high-dimensional feature $x\in R^{N}$ to a lower-dimensional space $v\in R^{M}$ as follows:

$$\begin{array}{c} v=Rx \end{array}$$ (8)

where *M* ≪ *N*. Each projection **v** is essentially identical to a compressive measurement during CS encoding. Therefore, using the CS principle, the number of data to be classified can be remarkably lower than the number of the original microarray gene data. With fewer data, the classification algorithm can also be more efficient. We refer to this reduction of microarray dimensionality as a CS dimensionality reduction.

Ideally, **R** can offer a stable embedding that approximately retains the important information in any *K*-sparse signal when $x\in R^{N}$ is projected to $v\in R^{M}$. Therefore, the so-called restricted isometry property (RIP) is derived to approximately maintain distances between any pair of *K*-sparse signals. Note that every signal pair shares the same *K* basis. That is, for any two *K*-sparse vectors *x*_1_ and *x*_2_ sharing the same K basis,

$$\begin{array}{c} \left(1-\epsilon \right)∥x_{1}-x_{2}{∥}_{2}^{2}\leq ∥Rx_{1}-Rx_{2}{∥}_{2}^{2}\leq \left(1+\epsilon \right){∥x_{1}-x_{2}∥}_{2}^{2} \end{array}$$ (9)

Incoherence is achieved if the sparse signal satisfies the RIP condition. For example, random matrices with entries identically and independently drawn from a standard normal distribution, Bernoulli distributions, or Fourier matrix satisfy the RIP condition. However, given that the matrix is dense, the loads in terms of memory and computational complexity become unacceptable when *N* is large. The matrix which satisfies the RIP condition can be directly obtained using the Johnson-Lindenstrauss lemma. In this paper, we use a very sparse random measurement matrix with entries defined as follows:

$$\begin{array}{c} r_{ij}=\sqrt{\rho }\times \{\begin{array}{cc} 1 & \text{with}\;\;\text{probability}\;\frac{1}{2\rho }\\ 0 & \text{with}\;\;\text{probability}\;\frac{1}{2\rho }\\ -1 & \text{with}\;\;\text{probability}\;\frac{1}{2\rho } \end{array} \end{array}$$ (10)

This type of matrix with *ρ* = 1 or 3 satisfies the Johnson-Lindenstrauss lemma [29]. This matrix is easy to compute and requires only a uniform random generator. In the latter approach, the microarray readouts are linear combinations of input gene expression data components. Thus, the readouts can be expressed in the form given by Eq 8. With a reduced number of measurements, we are able not just to detect but also classify the target gene expression datasets. The proposed CRC with CS dimensionality reduction algorithm is summarized in Algorithm 1.

**Algorithm 1** CRC Algorithm

**Input**: The gene expression sample datasets *D* = [*D*_1_, *D*_2_, ⋯, *D*_*K*_] and the test dataset y_*new*_.

**Output**: The classification result of *s*.

1: The dimensionality of *X* and y is reduced using the sensing matrix **R** in Eq 10. The new lower-dimensional versions of *D* and y_*new*_ are written as *X* and y, respectively.

2: The columns of *X* are normalized with *l*_2_-norm criteria.

3: y_*new*_ is coded over *X* by $\hat{\rho }=Py_{new}$ where *P* = (*X*^*T*^*X* + λ*I*)^−1^*X*^*T*^

4: The regularized residuals $r_{i}=\;∥y_{new}-X_{i}{\hat{\rho }}_{i}{∥}_{2}/{∥{\hat{\rho }}_{i}∥}_{2}$ are computed.

5: The identity of y_*new*_ is determinated as Identity(y_*new*_) = arg min_*i*_ *r*_*i*_

## Results and discussion

To evaluate the classification performance, we performed experiments on two benchmark datasets and compared the proposed model with state-of-the-art models. The simulations were conducted on a system with i7-4790 CPU with 3.60 GHz processor and 8 GB RAM. The algorithms were evaluated based on accuracy to determine the rating prediction accuracy as follows:

$$\begin{array}{c} Accuracy=\frac{TP+TN}{TP+FN+TN+FP} \end{array}$$ (11)

where TP denotes true positive (item is true and classified truly), TN is true negative (item is true but not classified truly), FP is false positive (item is false but classified truly), and FN is false negative (item is false and not classified truly).

The performance of the classifiers is quantified by LOO cross-validation (LOOCV) and 10-fold cross validation (10-fold-CV). In the LOOCV scheme, each sample in the dataset was predicted by building a model from the remaining samples and recording the accuracy of each model. In 10-fold-CV, the dataset was randomly divided into 10 equally sized subsets. Nine of these subsets were used in the model construction, while the remaining subset was used for prediction.

### Datasets

In this study, we use gene data for leukemia and ASD to evaluate the performance of CRC. The leukemia data set was downloaded from an open resource on the website of Gene Pattern in Broad Institute. The original training data set consisted of 38 bone marrow samples (27 ALL and 11 AML), while the testing data set consisted of 35 bone marrow samples (21 ALL and 14 AML) [14]. A total of 7129 gene expression samples were tested. The brain gene data was collected by a consortium consisting of Allen Institute for Brain Science and five collaborating universities [30–32]. Using the GENCODE consortium’s release [33], the expression data were assembled and aligned in the form of RNA-sequencing reads. The data were in the units of reads per kilobase of transcript per million mapped reads (RPKM). Therefore, A *log*_2_(RPKM+1) transformation operation was imposed on these data. The dataset had 524 samples with a developmental time point range from 8 weeks post-conception to 40 years of age from 26 brain structures. To our knowledge, this brain dataset is currently the most comprehensive transcriptome of the human developing brain. In the training dataset, the expression values for the temporospatial time points acted as features.

We also considered the diffuse large B-cell lymphoma (DLBCL) [34] and breast cancer, which has high dimension. The DLBCL data set consisted of 58 samples from DLBCL patients and 19 samples from follicular lymphoma with 7070 genes. The gene expression profiles were organized using Affymetrix human oligonucleotide arrays. The gene dataset of breast cancer has 14 docetaxel-resistant samples and 10 docetaxel-sensitive samples. The cDNA microarrays comprised 12625 genes. All datasets are downloaded from http://www.biolab.si/supp/bi-cancer/projections/index.html (Table 2), and have been preprocessed by *t*-test with a 0.05 confidence level.

**Table 2:** Gene data sets used in this study.

Data set	Samples	Genes	Classes
Leukemia	72	7129	2
DLBCL	77	7070	2
breast	24	12625	2
ASD	2128	524	2

### Dimensionality reduction results

**Fig 1:**
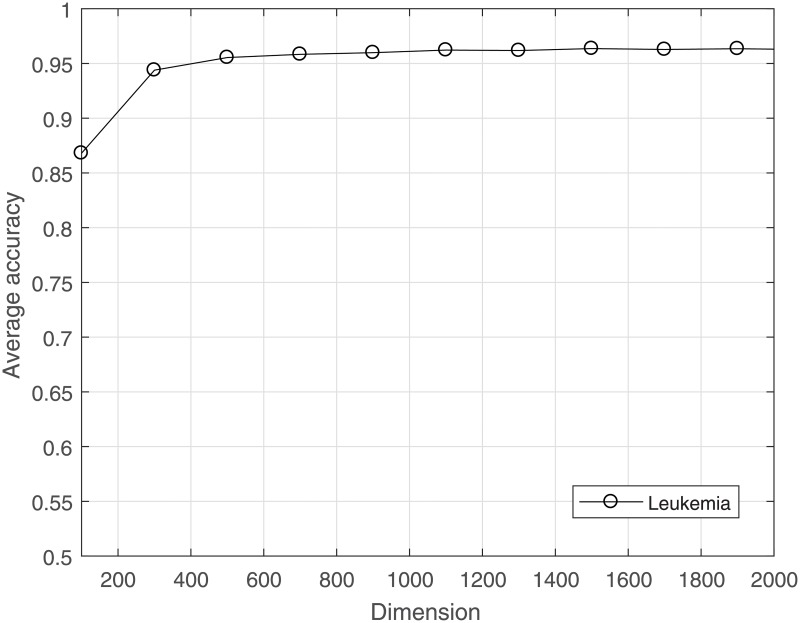
The average accuracy of CRC for leukemia dataset with reduced dimensionality.

**Fig 2:**
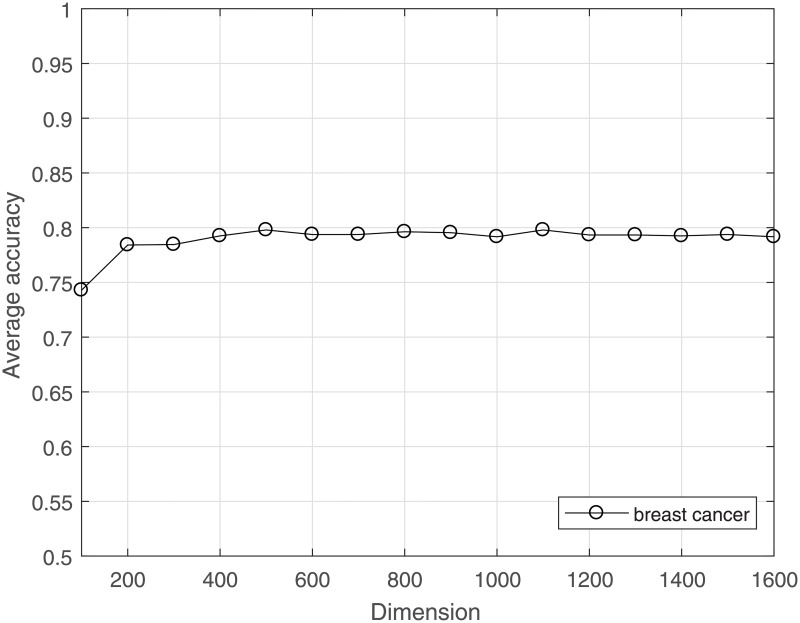
The average accuracy of CRC for breast cancer dataset with reduced dimensionality.

**Fig 3:**
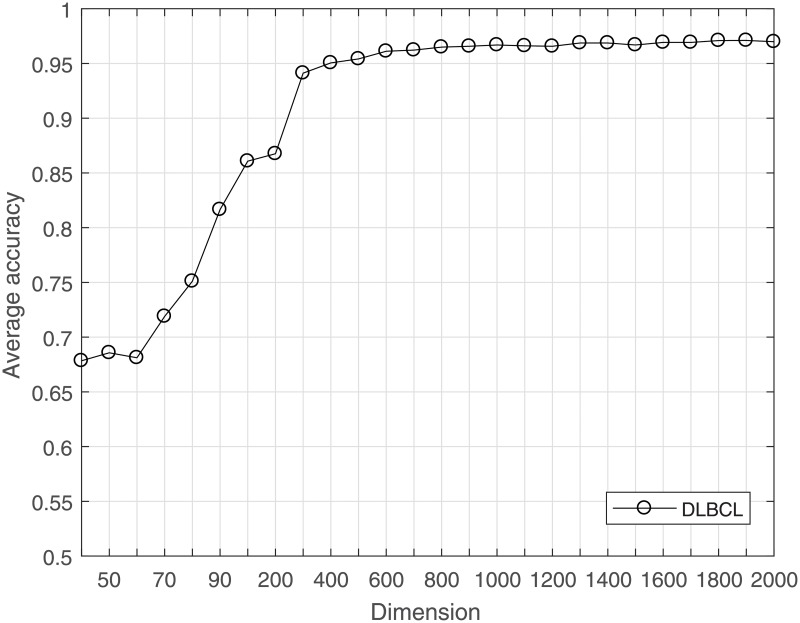
The average accuracy of CRC for DLBCL dataset with reduced dimensionality.

**Fig 4:**
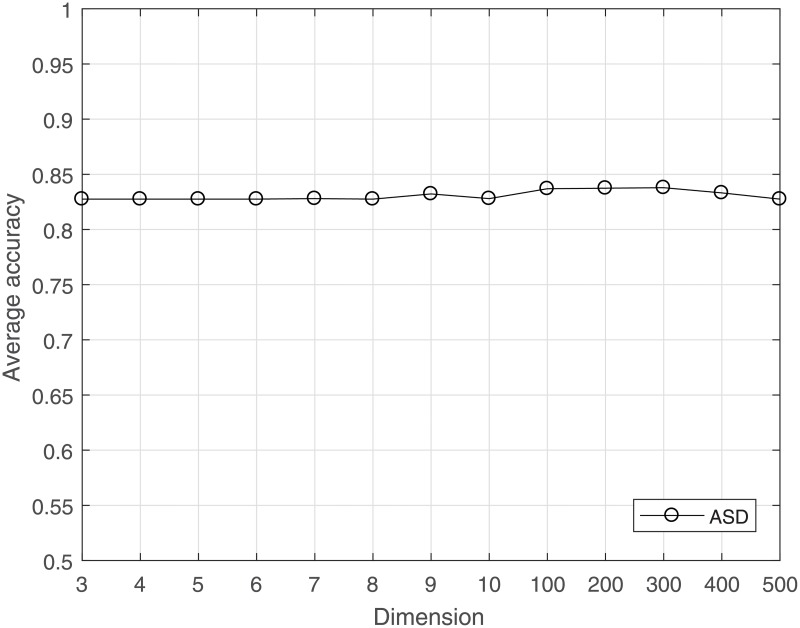
The average accuracy of CRC for ASD dataset with reduced dimensionality.

The computational load can be reduced, and overfitting can be avoided by reducing the dimensionality for a dataset. CS is used to project the high-dimensional data to a lower-dimensional space. The much lower bound for *M* is sufficient to achieve good results for gene data classification. The average LOOCV accuracy under the incremental dimension reduced by CS is shown in Figs 1–4. Fig 1 shows that in leukemia, when the reduced dimensionality is more than 500, the average accuracy would not increase. That is, 500 is an optimal choice for CS dimensionality reduction of leukemia gene data. Similarly, Figs 2 and 3 show that the optimal reduced dimensionalities for breast cancer and DLBCT are 200 and 600, respectively. By contrast, Fig 4 depicts the performance of ASD always remains stable even when the dimensionality is reduced to 3. Thus, the data in the gene datasets of this disease may be highly coherent. These coherent data may be sparse in the other form. Compared with the original dimensionality, CS dimensionality reduction is helpful to speed up the classification algorithm.

We also compared the running time of CRC and SRC with fast *l*_1_-minimization methods. We fixed the reduced dimensionality. In one LOOCV loop, the average running speeds of CRC and SRC are listed in Table 3.

**Table 3:** Running time of classification on the microarray database.

Data set	SRC	CRC	Best reduced dimension
Leukemia	0.55	0.56s	500
DLBCL	0.67	0.68s	600
Breast Cancer	0.4	0.4s	200
ASD	3.5	0.52s	3

The improved speed of CRC is obvious in the ASD database. However, in leukemia, DLBCL, and breast cancer datasets, the matrix *X* is overdetermined. SRC directly uses pseudoinverse to solve the *l*_1_ minimization problem. When more samples are used for training, SRC has to use the other fast *l*_1_ minimization methods, such as *l*_1_*ls* and homotopy. In such case, CRC is 7 times faster than SRC.

### Performance comparison

Several state-of-art classifier models, including SRC, KNN [35], support vector machines (SVM) [36], and random forest [37], were adopted when performances were compared in terms of averaged accuracy. The procedure was repeated 100 times, and their averaged performances were calculated for all tested samples to enhance the quantification of the performance of the learning model. The numerical results of the LOOCV, 10-fold CV, and 5-fold CV are summarized in Tables 4–6. For comparison, we also considered the small round blue cell tumors (SRBCT) which have multiple classes. Four different SRBCTs are used in this dataset, namely, Ewing family tumor (EWS), Burkitt lymphoma (BL), neuroblastoma (NB), and rhabdomysarcoma (RMS). The training set contained 63 samples, and the test set contained 20 samples. The cDNA microarrays consisted of 2308 genes.

**Table 4:** Leave-one-out cross validation (CV) classification results.

Data set	CRC	SRC	KNN	SVM	Random forest
Leukemia	96.5%	96.4%	84.7%	91.7%	90.0%
DLBCL	97.6%	95.5%	89.6%	84.4%	89.6%
Breast Cancer	79.2%	78.1%	74.4%	78.1%	68.1%
ASD	83.8%	82.1%	68.8%	71.4%	67.7%
SRBCT	98.9%	98.9%	97.8%	84.3%	92.8%

**Table 5:** 10-fold CV classification results.

Data set	CRC	SRC	KNN	SVM	Random forest
Leukemia	94.5%	94.1%	85.5%	91.6%	93.5%
DLBCL	94.5%	89.5%	89.6%	84.4%	89.6%
Breast Cancer	74.6%	73.1%	74.4%	73.1%	69.1%
ASD	82.8%	81.1%	70.8%	70.4%	68.7%
SRBCT	91.6%	91.6%	89.6%	84.8%	90.3%

**Table 6:** 5-fold CV classification results.

Data set	CRC	SRC	KNN	SVM	Random forest
Leukemia	87.5%	86.2%	80.2%	83.5%	86.5%
DLBCL	83.1%	80.5%	80.0%	79.5%	78.9%
Breast Cancer	70.8%	68.1%	68.4%	68.5%	67.1%
ASD	82.6%	80.9%	70.0%	70.4%	66.8%
SRBCT	73.6%	73.6%	70.6%	72.6%	73.1%

Tables 4 and 5 shows that CRC outperformed the other classifiers using all validation methods. This approach has an average accuracies of 96.4% and 94.5% using LOOCV and 10-fold-CV, respectively. The accuracies of SRC and CRC are quite close and at least 7% higher than those of the other three methods. Table 6 shows the performances in terms of 5-fold CV. All averaged accuracies are reduced compared with that of 10-fold CV. This phenomenon is due to the less samples used to train. Note that the performance of ASD was reduced slightly, because the total samples of ASD gene data were far more than the other gene datasets. The proposed method yielded a slightly higher average accuracy, implying that CR remarkably contributed to the classification. Considering these studies, CRC is the most robust method, because it showed the highest accuracy with the least number of genes not only in the test set but also using other types of validations, including 10-fold-CV and LOOCV.

## Conclusion

This paper presented a new classifier model for classifying microarray gene expression. The proposed classifier uniquely incorporates CS dimensionality reduction CS to guide data classification. This work has two important contributions. First, an effective CRC approach was implemented. This method exhibited very high performance and valuable insight into the different types of cancer data sets. CRC did not require the optimization of parameters to facilitate classification. This method can be used for different types of gene expression data with multiple classes without any modifications. Second, CS method was adopted to reduce the dimensionality of data. The sensing matrix is a very sparse random matrix with entries as 0 and $\sqrt{\rho }$. This method avoided the various feature extraction methods that are computationally expensive. The proposed method can be introduced for clinical application for each patient. Accurate diagnostics can be provided by only measuring a few gene expression data. We tested our algorithm on publicly available data sets of several diseases, including leukemia, breast cancer, ASD, DLBCL, and SRBCT. In conclusion, CRC can achieve high classification accuracy and fast computational speed.

## Supporting information

S1 File The code (Matlab) and data files along with instructions are provided as a zip file. (ZIP)
